# Chlorin e6-Mediated Photodynamic Therapy Suppresses *P*. *acnes*-Induced Inflammatory Response via NFκB and MAPKs Signaling Pathway

**DOI:** 10.1371/journal.pone.0170599

**Published:** 2017-01-24

**Authors:** Yoon-Young Wang, A-Reum Ryu, Solee Jin, Yu-Mi Jeon, Mi-Young Lee

**Affiliations:** 1 Department of Medical Science, College of Medical Sciences, Soonchunhyang University, Asan, Chungnam, Republic of Korea; 2 Korea Brain Research Institute, Research Division, Daegu, Republic of Korea; 3 Department of Medical Biotechnology, College of Medical Sciences, Soonchunhyang University, Asan, Chungnam, Republic of Korea; Massachusetts General Hospital, UNITED STATES

## Abstract

Photodynamic therapy (PDT), consisting of photosensitizer, light, and oxygen has been used for the treatment of various diseases including cancers, microbial infections and skin disorders. In this study, we examined the anti-inflammatory effect of chlorin e6-mediated PDT in *P*. *acnes*-infected HaCaT cells using photosensitizer chlorin e6 (Ce6) and halogen light. The live and heat-killed *P*. *acnes* triggered an upregulation of inflammatory molecules such as iNOS, NO, and inflammatory cytokine in HaCaT cells and mouse model. Ce6-mediated PDT notably downregulated the expression of these inflammatory molecules *in vitro* and *in vivo*. Similarly, chlorin e6-mediated PDT was capable of regulating inflammatory response in both live and heat killed *S*. *epidermidis* exposed HaCaT cells. Moreover, phosphorylation of p38, JNK, and ERK were reduced by Ce6-mediated PDT. Ce6-mediated PDT also reduced the phosphorylation of IKKα/β, IĸBα and NFκB p65 in *P*. *acnes*-stimulated HaCaT cells. In addition, the dramatic increase in the nuclear translocation of NFκB p65 observed upon stimulation with *P*. *acnes* was markedly impaired by Ce6-based PDT. This is the first suggestion that Ce6-mediated PDT suppresses *P*. *acnes*-induced inflammation through modulating NFκB and MAPKs signaling pathways.

## Introduction

Acne vulgaris is a common skin disease estimated to affect 660 million people in the world, making it the 8th most common disease worldwide (2013), ultimately affecting the quality of life in those with severe cases [1,2]. Acne-related skin inflammation is mediated by *Propionibacterium acnes (P*. *acnes)*' ability of activating a class of immune system and changing the sebum's lipid composition. The inflammatory cascade triggered by *P*. *acnes* leads to the formation of inflammatory lesions and scar formation [3,4].

Inflammation is a complex protective response against various harmful stimuli including pathogens and irritants. To abrogate the detrimental effects of inflammation, many researches looking at ways to regulate the inflammatory signaling pathways in various diseases, focusing on potential molecular targets for anti-inflammatory therapy, have been performed [5]. Inflammatory responses are mediated by multiple molecular mechanisms, and the most prominent is the production of pro-inflammatory cytokines and inflammatory molecules such as iNOS-derived NO [6,7]. It has been reported that a secreted peptidoglycan of *P*. *acnes* stimulates the production of various pro-inflammatory cytokines including IL-8 and TNF-α, thereby triggering inflammatory skin processes [8,9]. Generally, IL-8 activation, modulated by iNOS-derived NO, is involved in the recruitment of neutrophils, as well as activation of the MAPKs and NFκB signaling pathways [10].

Mitogen-activated protein kinases (MAPKs) play a crucial role in fundamental biological processes and cellular responses to external stressors. At least three MAPK families have been characterized: extracellular signal-regulated kinase (ERK), c-Jun N-terminal protein kinase (JNK) and p38 MAPK. MAPKs are potential therapeutic targets for anti-inflammatory response, because of their involvement in the regulation of inflammatory mediators at the transcriptional and translational levels [11,12].

Activated MAPKs mediate the signaling cascades leading to activation of various transcription factors such as nuclear factor-kappa B (NFκB) [13,14]. NFκB is a principal transcription factor playing an important role in inflammation and immune response [15]. NFκB dimers are located in the cytoplasm through interaction with inhibitory proteins IκBs in non-stimulated cells. However, upon stimulation mainly with pro-inflammatory cytokines, activated protein kinase I kappa B kinase (IKK) phosphorylates IκBs. Phosphorylated IκBs are subsequently ubiquitinated and degraded. As a consequence, free NFκB dimer, which is spared from degradation, enters the nucleus and activates transcription of a variety of genes including pro-inflammatory genes [15–17]. Anti-inflammatory agents targeting the MAPK and NFκB pathways, primarily via suppression of the expression of inflammatory mediators, have been developed [13,14,18].

Photodynamic therapy (PDT), based on a photosensitizer, light source, and molecular oxygen, has been used clinically to treat a wide variety of diseases such as cancers and non-neoplastic diseases with minimal side effects [19–21]. Especially, PDT is becoming more widely recognized as a valuable treatment option for localized cancers, as technology for new photosensitizers and light sources are developed and applied. Cytotoxic singlet oxygen and other reactive oxygen species (ROS) are produced when the light-absorbing compound, the photosensitizer, is illuminated with light of a specific wavelength in the presence of molecular oxygen, the principle upon which PDT exerts its clinical effect [22,23].

Among several natural and synthetic PDT photosensitizers, chlorin e6 (Ce6) is a promising second generation photosensitizer with high efficacy and minimal toxicity [24]. Especially, chlorin e6 was a much stronger antibacterial photosensitizer than 5-aminolevulinic acid, generally used in clinical PDT [25]. The illumination with specific wavelengths of light is required for inducing the phototoxic effect of photosensitizer in PDT. Although laser light is the most widely used light source for PDT, there are a number of difficulties that must be overcome in the safety. Thus, selection of safer and more efficient light source is important in PDT. We have used Ce6 associated with halogen light in PDT to investigate the feasibility of therapeutic potential against acne in our previous report [25]. Ce6-mediated PDT with halogen light showed superior therapeutic potential probably due to its anti-microbial, anti-oxidative and anti-inflammatory effects. However, the underlying molecular mechanism by which Ce6-mediated PDT exerted its anti-inflammatory effect has not yet been elucidated.

In this investigation, we examined the suppressive effect of Ce6-mediated PDT on the production of inflammatory molecules and its relevance in relation to the NFκB and MAPKs signaling pathways in *P*. *acnes*-treated HaCaT cells. The inhibitory effect of Ce6-mediated PDT on inflammatory molecule generation via NFκB and MAPKs inhibition may emerge as an attractive strategy to treat inflammatory diseases caused by microbial infections.

## Materials and Methods

### Bacterial culture

*P*. *acnes* was obtained from the Korean collection for type cultures (KCTC) and grown under anaerobic conditions in 100 ml of reinforced clostridial solid and liquid medium (RCM) (Difco Laboratories, Detroit, MI) at 37°C for 3 days in order to reach stationary phase (bacterial concentration was 10^9^ CFU/ml) at which time the bacteria were harvested. *Staphylococcus epidermidis* (*S*. *epidermis*) obtained from KCTC was cultured at 37°C for 24 h with nutrient broth under aerobic conditions. The bacteria were harvested via centrifugation of the cultures at 6,000 rpm for 30 min at 4°C. The resulting bacterial pellets were pooled and washed in cold PBS and centrifuged again. Finally, the pooled bacterial pellet was resuspended in PBS. For obtaining heat-killed bacteria, the bacterial suspension was heated at 80°C and the supernatant was removed by centrifugation at 10,000 rpm for 20 min at 4°C. This processed pellet was used for the stimulation experiments.

### Chlorin e6-mediated photodynamic therapy

The human keratinocyte cell line HaCaT was purchased from the American Type Culture Collection (ATCC). The cells were grown in Dulbecco’s modified Eagle’s medium (DMEM) (Hyclone Labs., Logan, USA) supplemented with 10% heat-inactivated fetal bovine serum (FBS) (Hyclone Labs., Logan, USA), 100 U/ml penicillin and 100 μg/ml streptomycin at 37°C in a humidified atmosphere with 5% CO_2_. For the stimulation experiment, HaCaT cells were incubated with live or heat-killed *P*. *acnes* adjusted at the appropriate concentration in serum free media for 24 h at 37°C in 5% CO_2._ After stimulation, the HaCaT cells treated with or without Ce6 for 30 min at 37°C in 5% CO_2_. After which the HaCaT cells were illuminated with halogen lamp at 5,000 lx for 30 min. Ce6 was synthesized from *Spirulina* chlorophyll as shown in our previous report [25]. The animal study involving live and heat-killed *P*. *acnes* was performed in accordance to the mouse inflammation model of our previously published report [25].

### No production measurement

The nitrite concentration in conditioned medium was measured as an indicator of NO production according to the Griess reaction. Each supernatant (100 μl) was mixed with the same volume of Griess reagent (1% sulfanilamide in 5% phosphoric acid and 0.1% naphthylethylenediamine dihydrochloride in distilled water); the absorbance of the mixture was determined with an ELISA reader (Sunrise, Tecan, Switzerland) at 570 nm.

### Cytokine measurement

The effect of Ce6-mediated PDT on the production of IL-8 in heat-killed *P*. *acnes*-infected HaCaT cells were measured by ELISA. The cytokine concentrations were calculated according to the standard curve using recombinant cytokines in the ELISA kit.

### Western blotting

The protein was separated by 10% SDS‐PAGE, and then transferred onto polyvinylidene fluoride membrane (Bio-Rad Laboratories, Inc. Hercules, CA). The membranes were incubated overnight at 4°C with primary antibodies [iNOS (Abcam Ltd, Cambridge, UK), IKKα/β, p-IKKα/β, IκBα, p-IκBα, NFκB p65, p-NFκB p65, p38, p-p38, ERK, p-ERK, JNK, p-JNK (Cell Signaling Technology, Inc., MA, USA) and β-actin (Santa Cruz Biotechnology, Inc., Dallas, TX, USA)] which were diluted follow manufacturer’s recommendation. The membranes were then washed in TBST and incubated with the appropriate secondary antibody HRP-conjugated (1:5000) at room temperature for 1 h. The separated proteins were visualized using the ECL plus Western blotting detection system. The intensity of the bands was analyzed using ImageQuant TL software (GE Healthcare Bio-Science Corp., Piscataway, NJ) [26,27].

### Confocal microscope analysis

Heat-killed *P*. *acnes*-infected HaCaT cells were treated with Ce6-mediated PDT, after which the cells were fixed with 4% paraformaldehyde in PBS for 20 min and permeabilized with 0.5% Trixon X-100 for 15 min. After 1 h incubation with blocking buffer (5% BSA in PBS), the cells were incubated with primary antibodies (NFκB p65 Rabbit mAB) (1:100) (Cell Signaling Technology, Inc., MA, USA) in 0.5% BSA for overnight at 4°C. The cells were washed in PBS for 10 min, done 3 times, and were stained for another 1 h with goat anti-rabbit IgG Texas red (1:1000) (Santa Cruz Biotechnology, Inc., USA). Nuclei were counterstained with 4, 6-diamidino-2-phenylindole dihydrochloride (DAPI) (Bio-Rad, USA). The prepared cells were then observed under a fluorescent microscope and images were recorded.

### Statistical analysis of data

Data were entered into a Microsoft Excel datasheet and then transferred into the Statistical Package for Social Sciences (SPSS, version 18). Data from each group are expressed as mean ± SEM. Group differences were analyzed using one-way analysis of variance (ANOVA). *P*<0.05 was considered statistically significant.

## Results

### Inhibitory effects of Ce6-mediated PDT on live and heat-killed *P*. *acnes*-induced inflammation in HaCaT cells

**Fig 1:**
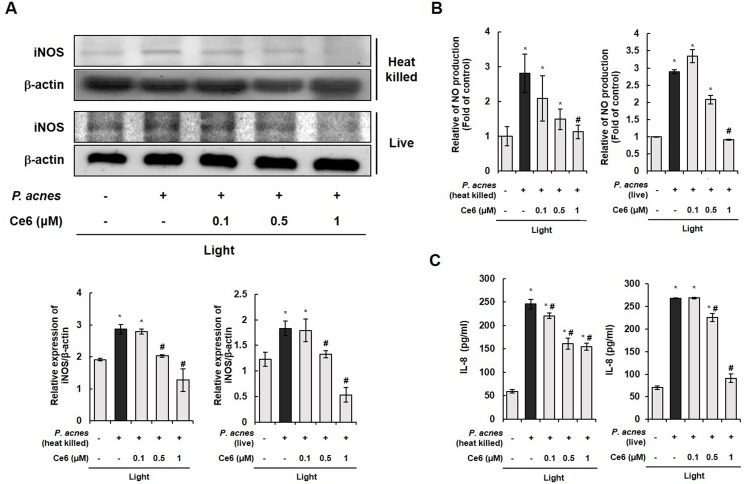
Ce6-mediated PDT effectively inhibited iNOS, NO, and inflammatory cytokine IL-8 in *P*. *acnes*-triggered inflammation (A) Western blot results demonstrate that Ce6-mediated PDT suppressed the expression iNOS in live and heat-killed *P*. *acnes*-infected HaCaT cells. The ratio of immunointensity between the iNOS and β-actin was calculated. (B) Ce6-mediated PDT suppressed NO production in live and heat-killed *P*. *acnes*-stimulated HaCaT cells. (C) ELISA results demonstrate that Ce6-mediated PDT reduced IL-8 in live and heat-killed *P*. *acnes*-infected HaCaT cells. Results are expressed as mean ± SEM. *P<0.05 compared with the non-treated control group. ^#^*P*<0.05 compared with *P*. *acnes* treated cells only.

The anti-inflammatory activity of Ce6-mediated PDT was examined by investigating it’s effect on live and heat-killed *P*. *acnes*-induced iNOS expression through western blotting (Fig 1A). The level of iNOS protein was notably increased in HaCaT cells upon exposure to live *P*. *acnes*. However, Ce6-mediated PDT using halogen light suppressed the expression of iNOS at 0.5 and 1 μM Ce6. The result indicated that live *P*. *acnes* induced an inflammatory response via enhancing iNOS level, but Ce6-mediated PDT inhibited the expression of iNOS. The effect of Ce6-mediated PDT on the production of inflammatory mediator NO was assessed by measuring the accumulation of nitrite. Nitrite level in the culture medium was monitored by Griess reagent assay in cells stimulated with live *P*. *acnes* with or without the application of Ce6-mediated PDT. Live *P*. *acnes* induced a significant increase in NO production in HaCaT cells as compared to the control group. Ce6-mediated PDT significantly suppressed the production of NO in live *P*. *acnes*-infected HaCaT cells (Fig 1B). Taken together, Ce6-mediated PDT inhibited the expression of iNOS enzymes, which subsequently reduced the production of NO, a key mediator of inflammatory response. The concentration of secreted inflammatory cytokine IL-8 in live *P*. *acnes*-infected HaCaT cells were measured by using ELISA. As shown in Fig 1C, live *P*. *acnes* triggered IL-8 secretion in HaCaT cells, but the IL-8 secretion was reduced by Ce6-mediated PDT in these cells.

Interestingly, similar results were obtained with regards to iNOS, NO and IL-8 levels in HaCaT cells exposed to either heat-killed *P*. *acnes* or live *P*. *acnes*. Taken together, Ce6-mediated PDT was capable of regulating inflammatory response in live and heat killed *P*. *acnes*-infected HaCaT cells.

**Fig 2:**
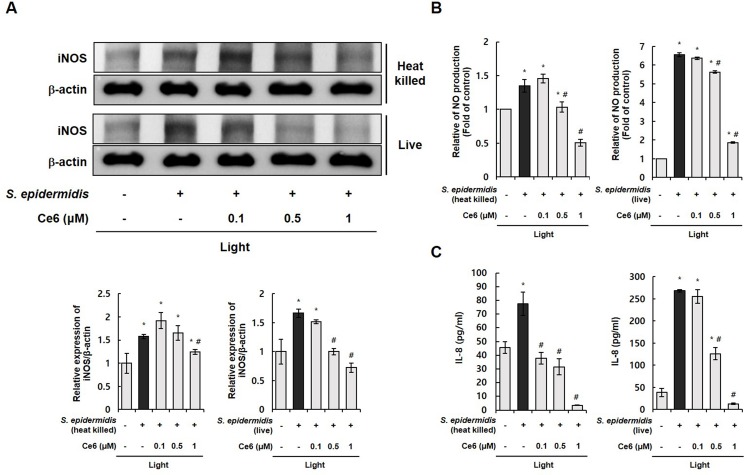
Ce6-mediated PDT effectively inhibited iNOS, NO, and inflammatory cytokine IL-8 in *S*. *epidermidis*-triggered inflammation (A) Western blot results demonstrate that Ce6-mediated PDT suppressed the expression iNOS in live and heat-killed *S*. *epidermidis*-infected HaCaT cells. The ratio of immunointensity between the iNOS and β-actin was calculated. (B) Ce6-mediated PDT suppressed NO production in live and heat-killed *S*. *epidermidis*-stimulated HaCaT cells. (C) ELISA results demonstrate that Ce6-mediated PDT reduced IL-8 in live and heat-killed *S*. *epidermidis*-infected HaCaT cells. Results are expressed as mean ± SEM. *P<0.05 compared with the non-treated control group. ^#^*P*<0.05 compared with *S*. *epidermidis* treated cells only.

Various skin microflora, including *P*. *acnes* and *S*. *epidermidis*, can co-exist in acne lesions [28]. Thus, another skin flora, *S*. *epidermidis*, was used to examine the PDT effect of Ce6 and halogen light against acne. Fig 2 shows that live and heat-killed *S*. *epidermidis* triggered an upregulation of inflammatory molecules such as iNOS, NO, and inflammatory cytokine IL-8 in HaCaT cells. Ce6-mediated PDT notably downregulated the expression of these inflammatory molecules. These results suggest that Ce6-mediated PDT effectively inhibited various inflammatory molecules induced by *S*. *epidermidis* and *P*. *acnes* which can co-exist in acne lesions.

### Inhibitory effects of Ce6-mediated PDT on live and heat-killed *P*. *acnes*-induced inflammation in mouse model

**Fig 3:**
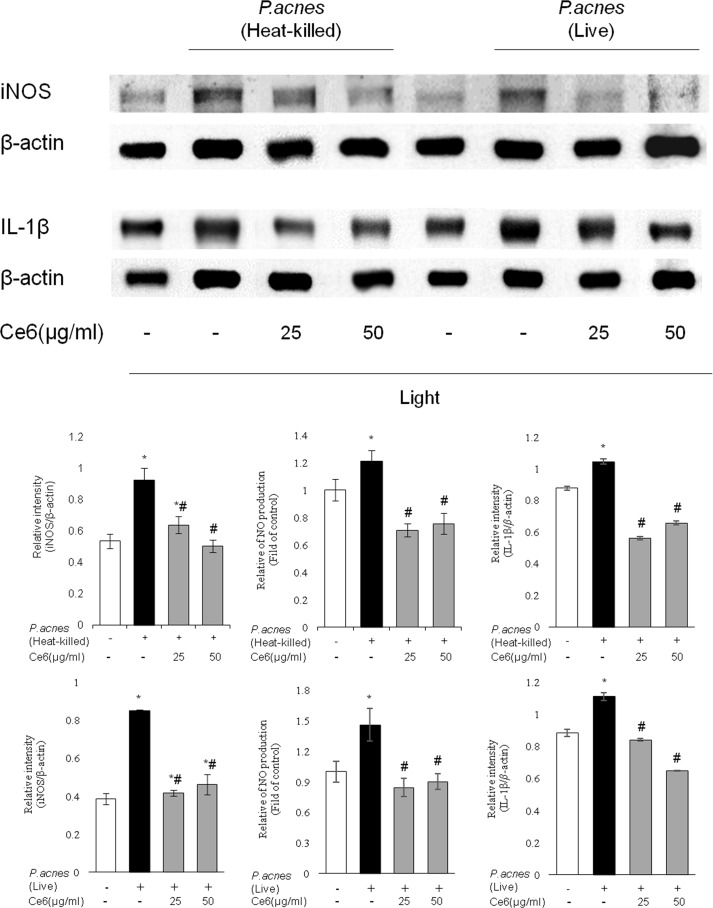
*In vivo* therapeutic effects of Ce6-mediated PDT on live and heat-killed *P*. *acnes*-induced inflammation. The left ear of ICR mice was injected with live and heat-killed *P*. *acnes*. Right ear received an equal amount of PBS serving as a control. Ce6 (1, 25 and 50 μg/ml in PBS) was epicutaneously applied on the left ear. Ce6-mediated PDT markedly decreased the expression of iNOS, NO and pro-inflammatory cytokine IL-1β induced by live and heat-killed *P*. *acnes*. Results are expressed as mean ± SEM. *P<0.05 compared with the non-stimulated control group. ^#^*P*<0.05 compared with *P*. *acnes* stimulated mice only.

*In vivo* anti-inflammatory effects of Ce6-mediated PDT with halogen light on the levels of inflammatory molecules, such as iNOS, NO and IL-1β, in mouse model were examined and shown in Fig 3. Intradermal injection with live *P*. *acnes*-alone enhanced the level of iNOS, NO and IL-1β compared to the control. The elevated levels of these inflammatory molecules were notably reduced upon applying Ce6-mediated PDT. Moreover, the application of Ce6-based PDT also significantly reduced heat-killed *P*. *acnes*–induced inflammatory molecules. These results show that Ce6-mediated PDT effectively blunted the elevation of inflammatory molecules induced by live and heat-killed *P*. *acnes* in mouse model.

### Effects of Ce6-mediated PDT on NFκB and MAPK signals in *P*. *acnes*-stimulated HaCaT cells

**Fig 4:**
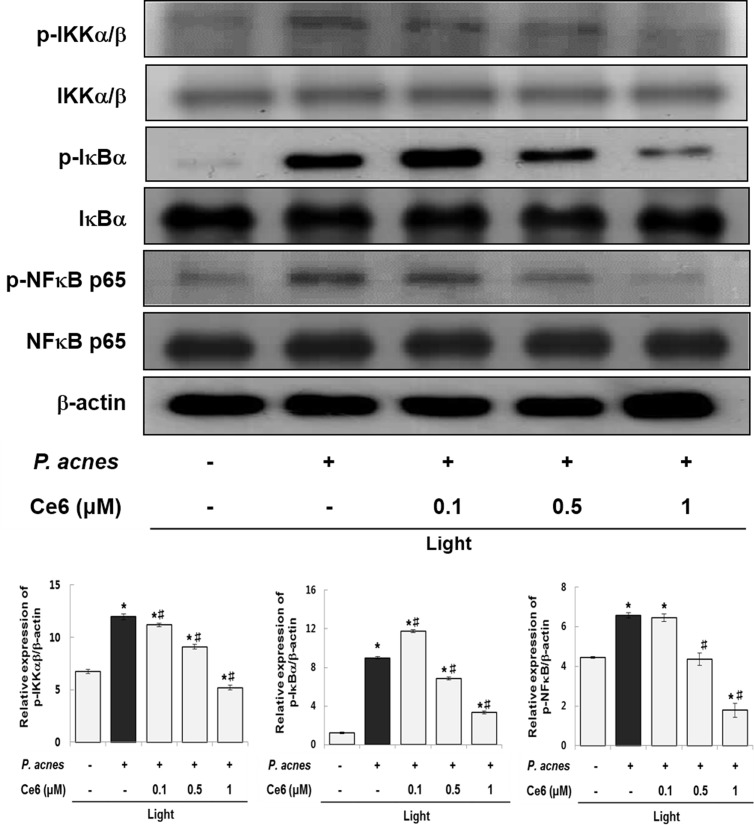
Ce6-mediated PDT suppressed NFκB signaling pathways in heat-killed *P*. *acnes*-stimulated HaCaT cells. Western blot analysis shows that phosphorylation of IKKα/β, IκBα and NFκB were suppressed by Ce6-mediated PDT. Results are expressed as mean ± SEM. *P<0.05 compared with the non-treated control group. ^#^*P*<0.05 compared with the heat-killed *P*. *acnes* treated cells only.

The expression pattern of NFκB-related proteins was examined by western blotting in order to determine whether the NFκB pathway is involved in the anti-inflammatory capability of Ce6-mediated PDT (Fig 4). The expression of phosphorylated IKKα/β, IκBα and NFκB p65 were upregulated in HaCaT cells in response to *P*. *acnes* stimulation. However, Ce6-mediated PDT markedly down-regulated the induced expression of phosphorylated IKKα/β, IκBα and NFκB. These results showed that treatment with Ce6-mediated PDT abolished the *P*. *acnes*-induced expression of NFκB-related proteins.

**Fig 5:**
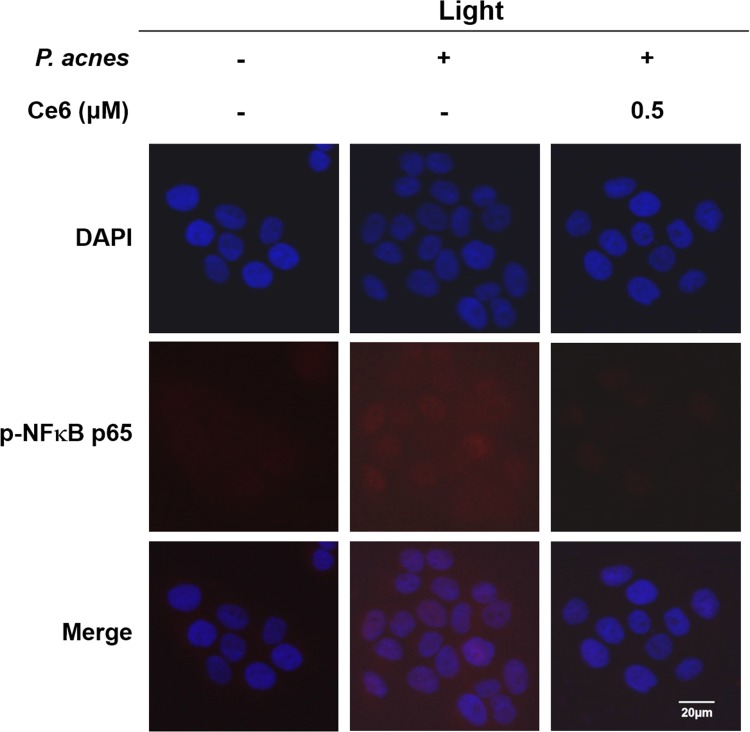
Effect of Ce6-mediated PDT on NFκB p65 translocation in heat-killed *P*. *acnes*-infected HaCaT cells. Representative photomicrographs of immune-labelled NFκB (red) and nuclear counterstaining with DAPI (blue) in *P*. *acnes*-exposed HaCaT cells without and with 0.5 μM Ce6 mediated PDT. *P*. *acnes* infection induced increased co-localization of NFκB and DAPI (purple) in HaCaT cells. However, Ce6 mediated PDT reduced the nuclear translocation of NFκB. Scale bar = 20 μm.

There was evidence that nuclear translocation of NFκB p65 occurred in *P*. *acnes*–infected cells and this nuclear translocation of NFκB was notably impaired after Ce6 treatment with halogen light illumination in Fig 5. Upon treating 0.5 μM Ce6 with halogen light, nuclear translocation and accumulation of NFκB p65 was dramatically inhibited.

**Fig 6:**
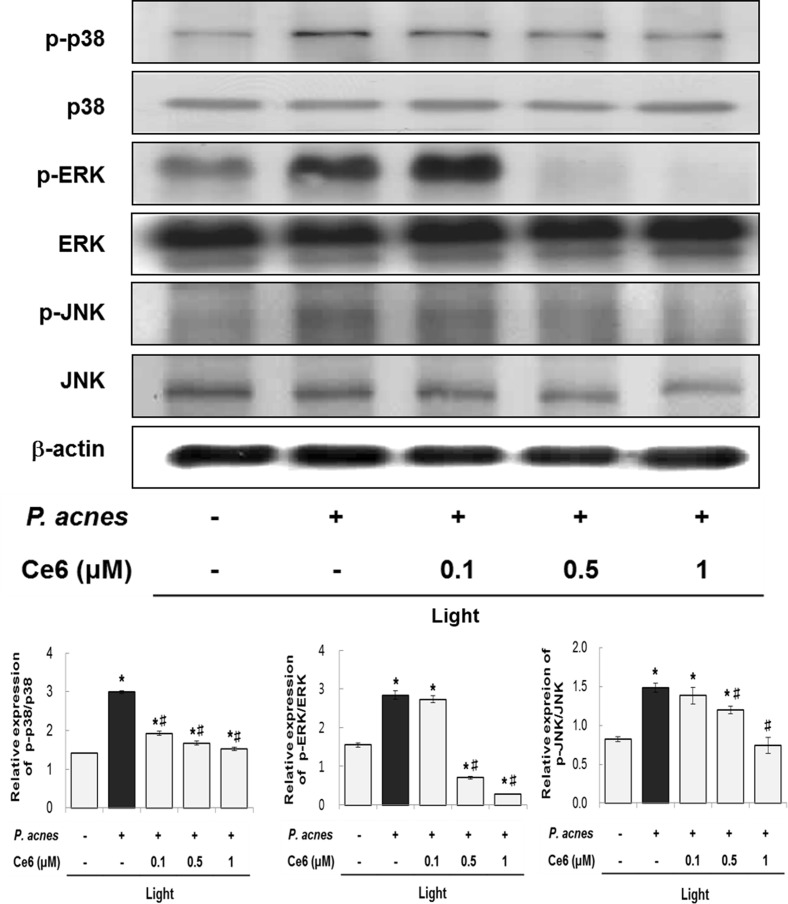
Effects of Ce6-mediated PDT on MAPKs signaling pathways in heat-killed *P*. *acnes*-infected HaCaT cells. Western blot analysis shows that the phosphorylation of p38, JNK and ERK were suppressed by Ce6-mediated PDT. Results are expressed as mean ± SEM. *P<0.05 compared with the non-treated control group. ^#^*P*<0.05 compared with the heat-killed *P*. *acnes* treated cells only.

MAPK activation is involved in the modulation of inflammatory response [6,29]. In Fig 6, we examined the effect of Ce6-mediated PDT on MAPK signals in *P*. *acnes*-induced inflammatory response in HaCaT cells. The phosphorylation of p38, ERK and JNK in these cells were significantly enhanced after treatment with heat-killed *P*. *acnes*. In contrast, phosphorylation of p38, ERK and JNK were reduced upon applicating Ce6-mediated PDT. More specifically, the phosphorylation of p38, ERK and JNK were prominently inhibited at 0.5 and 1 μM Ce6-mediated PDT. These results demonstrated that Ce6-mediated PDT exerted anti-inflammatory effect via modulating the NFκB and MAPK pathway.

## Discussion

The bacteria, derived from acne patients, that contribute to the inflammatory pathogenesis in acne include *P*. *acnes*, *S*. *epidermidis*, and *S*. *aureus*, etc. *P*. *acnes*, a gram-positive anaerobic bacterium located in pilosebaceous folicles of the skin, play a pivotal role in the development of inflammatory skin diseases such as acne vulgaris [30]. Antibiotics have been commonly used to treat infections caused by *P*. *acnes*, mainly due to the susceptibility of *P*. *acnes* to a broad range of antibacterial agents. However, the emergence of antibiotic-resistant skin flora represents a growing problem globally [31,32].

Recently, PDT, a clinically approved non-surgical therapeutic alternative for cancers and non-neoplastic diseases, has been developed as an alternative therapy for the treatment of acne, capable of overcoming antibiotic resistance [33]. Moreover, PDT has offered better patient compliance and high efficacy with lower risk [33,34]. However, the information on the molecular and cellular mechanism which underlies the clinical therapeutic efficacy of PDT is limited.

A remarkable therapeutic effect of Ce6-mediated PDT with halogen light against acne was already demonstrated in our previous report [25]. However, the information on the underlying molecular mechanism of Ce6-mediated PDT’s anti-inflammatory capability against *P*. *acnes*-induced inflammation is not available.

Among the different signaling components of the NFκB pathway, the most prominent transcriptional factor of NFκB is the p65/p50 heterodimer [15]. The IκB kinase (IKK) phosphorylates the inhibitory IκBα protein, which results in the dissociation of IκBα from NFκB. The free NFκB is then translocated into the nucleus and activates the expression of at least 150 genes that participate in inflammation signaling [16,17].

We got the same anti-inflammatory PDT phenomenon in HaCaT cells co-cultured with live and heat-killed *P*. *acnes* as well as in HaCaT cells co-cultured with live and heat-killed *S*. *epidermis*, as shown in Fig 1 and Fig 2, respectively. In addition, the anti-inflammatory PDT effect was also shown in animal inflammation model triggered by live and heat-killed *P*. *acnes* (Fig 3). These results suggest that similar inflammatory pathway occurred in live and heat-killed bacteria triggered inflammation. The anti-inflammatory effect of Ce6-mediated PDT may be broadly effective against various cutaneous resident flora associated acne, and not limited to a specific bacteria like *P*. *acnes*.

The anti-inflammatory properties of Ce6-mediated PDT might be due to its effect upon the NFκB pathway, where in several proteins in this pathway were activated, as shown in Fig 4. Increased expression of phosphorylated IKK, IκBα, and NFκB were found in HaCaT cells exposed to heat-killed *P*. *acnes*. However, treatment with Ce6-based PDT dramatically suppressed the phosphorylation of IKK, IκBα and NFκB. Moreover, the prominent nuclear accumulation of NFκB p65 seen in *P*. *acnes*–infected HaCaT cells were dramatically impaired with Ce6-mediated PDT, indicating suppression of the nuclear translocation of NFκB (Fig 5). These results demonstrated that treatment with Ce6-mediated PDT attenuated the effect of *P*. *acnes* on HaCaT cells by modulating NFκB signaling proteins.

MAPKs pathway is also associated with the modulation of pro-inflammatory mediator expression [13,14]. Three MAPKs; ERK, p38 and JNK, are known to be activated by several external stimulants including ROS and microbial infection [35,36]. MAPKs play an important role in the transcriptional regulation of iNOS, COX-2 and inflammatory cytokines via activation of the transcription factor NFκB in various cells [37–39]. In this investigation, the phosphorylated p38, ERK and JNK significantly increased in heat-killed *P*. *acnes*-infected cells, however, phosphorylated p38, ERK and JNK were dramatically reduced after Ce6-mediated PDT treatment as shown in Fig 6.

**Fig 7:**
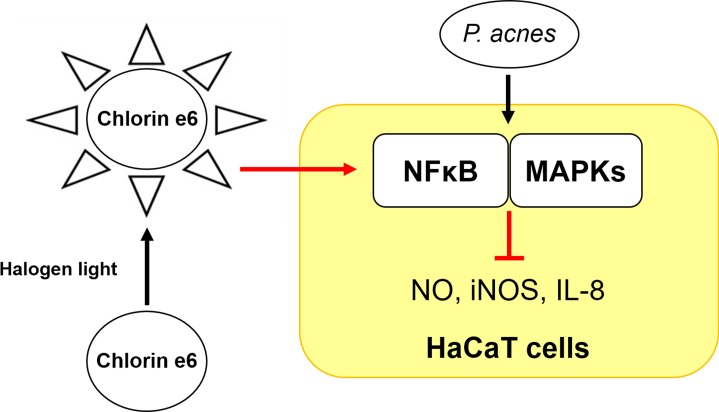
Schematic diagram of the suppressive effect of chlorin e6-based photodynamic therapy on the inflammatory response via NFκB and MAPKs in *P*. *acnes*-infected HaCaT cells.

These results demonstrate that Ce6-mediated PDT acted as a potent anti-inflammatory therapy by inhibiting *P*. *acnes*-mediated iNOS, NO and IL-8 production via suppressing the NFκB and MAPKs activation pathways (Fig 7). These results also suggest the feasibility of Ce6-mediated PDT for the treatment of *P*. *acnes*–induced inflammatory skin diseases.

## Supporting Information

S1 Table Antibody information. (PDF)
