# Supplementary material for: Chlorin e6-Mediated Photodynamic Therapy Suppresses P. acnes-Induced Inflammatory Response via NFκB and MAPKs Signaling Pathway
Source: PLoS One. 2017 Jan 24;12(1):e0170599. doi: 10.1371/journal.pone.0170599 (PMC5261614; doi:10.1371/journal.pone.0170599)
Supplement: S1 Table — (PDF) [file pone.0170599.s001.pdf]

Protein target	Manufacturer	Cat. No.	Species raised in; monoclonal or polyclonal	Dilution used
iNOS	Abcam	Ab3523	Rabbit, polyclonal	1:1000
IKKα/β	Cell Signaling Technology	2370	Rabbit, monoclonal	1:1000
p- IKKα/β	Cell Signaling Technology	2697	Rabbit, monoclonal	1:1000
IκBα	Cell Signaling Technology	4814	Mouse, monoclonal	1:1000
p-IκBα	Cell Signaling Technology	9246	Mouse, monoclonal	1:1000
NFκB p65	Cell Signaling Technology	6956	Mouse, monoclonal	1:1000
p-NFκB p65	Cell Signaling Technology	3033	Rabbit, monoclonal	1:1000
P38	Cell Signaling Technology	9212	Rabbit, polyclonal	1:1000
p-P38	Cell Signaling Technology	9211	Rabbit, polyclonal	1:1000
ERK	Cell Signaling Technology	4695	Rabbit, polyclonal	1:1000
p-ERK	Cell Signaling Technology	9101	Rabbit, polyclonal	1:1000
JNK	Cell Signaling Technology	9252	Rabbit, polyclonal	1:1000
p-JNK	Cell Signaling Technology	9251	Rabbit, polyclonal	1:1000
β-actin	Santa Cruz Biotechnology	sc-47778	Mouse, monoclonal	1:5000
